# Artificial Intelligence Reporting Guidelines in Dentomaxillofacial Radiology: Specialty-Oriented Practical Guide

**DOI:** 10.3390/diagnostics16182961

**Published:** 2026-09-13

**Authors:** Elaine Dinardi Barioni, Lays Assolini Pinheiro de Oliveira, Marcos Murilo Alves Santos, Kaan Orhan, Sérgio Lúcio Pereira de Castro Lopes, Andre Luiz Ferreira Costa

**Affiliations:** 1 Postgraduate Program in Dentistry, Dentomaxillofacial Radiology and Imaging Laboratory, Cruzeiro do Sul University (UNICSUL), São Paulo 01506-000, SP, Brazil; elainedinardi2@gmail.com (E.D.B.);; 2 Department of Diagnosis and Surgery, The Institute of Sciences and Technology, São Paulo State University (UNESP), São José dos Campos 12245-000, SP, Brazil; marcos.murilo@unesp.br (M.M.A.S.);; 3 Department of Dentomaxillofacial Radiology, Faculty of Dentistry, Ankara University, Ankara 06560, Turkey; 4 Department of Anesthesiology, Oncology and Radiology, Faculty of Medical Sciences, University of Campinas (UNICAMP), Campinas 13083-887, SP, Brazil

**Keywords:** artificial intelligence, cone-beam computed tomography, diagnostic imaging, radiomics, reporting guidelines

## Abstract

Artificial intelligence (AI) is becoming an increasingly important component of dentomaxillofacial radiology, with applications ranging from panoramic radiography and cone-beam computed tomography (CBCT) to magnetic resonance imaging, radiomics, and other emerging AI approaches. As AI studies become more methodologically complex, concerns regarding transparency, reproducibility, validation, and clinical applicability have also increased. Several reporting resources, including reporting guidelines, methodological quality assessment tools, and broader principles for trustworthy AI, have been developed to improve the quality and transparency of AI research. However, selecting the most appropriate resource remains challenging, particularly for studies that combine radiomics, predictive modeling, diagnostic accuracy assessment, segmentation, multimodal AI, and clinical validation. This specialty-oriented practical guide provides an overview of the main AI reporting resources and offers practical recommendations to support their selection and complementary use in dentomaxillofacial radiology. Common study designs, methodological challenges, and reporting considerations are discussed, together with practical guidance for readers and reviewers. The complementary roles of different reporting resources are illustrated through tables, decision aids, conceptual figures, and examples from the dentomaxillofacial imaging literature. By promoting more transparent and consistent reporting practices, this guide aims to support the development of more reproducible, clinically relevant, and trustworthy AI research in dentomaxillofacial radiology.

## 1. Introduction

The increasing adoption of artificial intelligence (AI) applications in medical imaging has been accompanied by increasing concerns regarding transparency, reproducibility, and methodological consistency in published research. As AI-based studies become progressively more usual across imaging specialties, the need for clear and standardized reporting has become increasingly evident. In response, several reporting guidelines have been developed to improve the description of AI methodologies, facilitate peer review, and support the responsible translation of algorithms into clinical practice. Rather than functioning simply as publication checklists, these frameworks aim to ensure that AI studies are reported with sufficient clarity to allow critical appraisal, reproducibility, and meaningful comparison across investigations [1,2].

Recognizing the diversity of AI study designs, Klontzas et al. [3] proposed a pragmatic framework to assist researchers in selecting the most appropriate reporting guideline according to the objectives and methodological characteristics of a given investigation. Their work highlighted that no single framework is universally sufficient and that the complementary use of multiple guidelines is often necessary. However, the proposed framework was intentionally broad, focusing on medical imaging research as a whole rather than on the specific methodological challenges encountered in individual imaging subspecialties [3].

**Figure 1:**
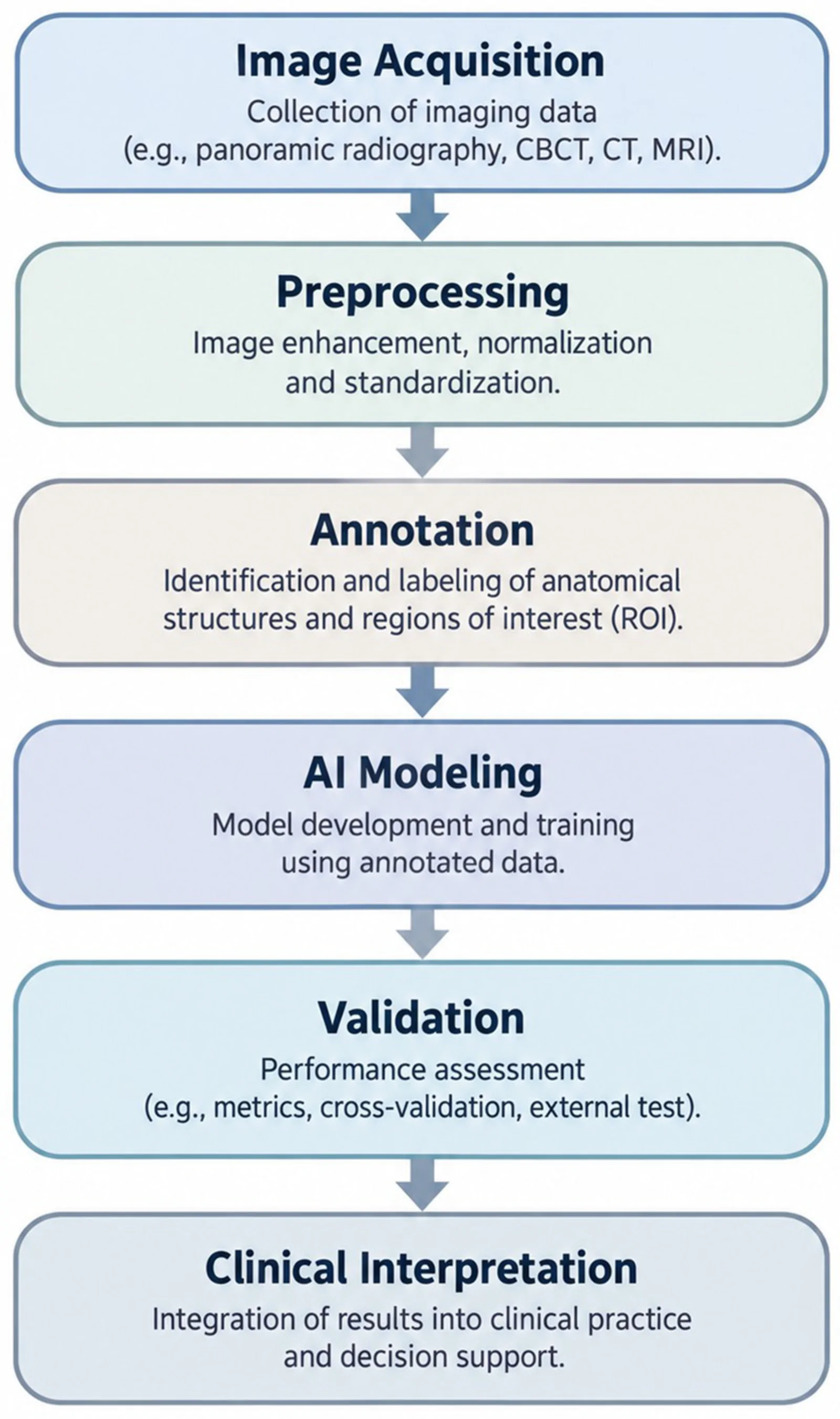
General workflow of AI studies in dentomaxillofacial radiology. The figure illustrates the main stages of an AI imaging study, from image acquisition and preprocessing to annotation, AI modeling, validation, and clinical interpretation. This conceptual workflow provides the methodological context for the reporting resources discussed throughout this guide.

Therefore, this article provides a specialty-oriented practical guide that adapts the guideline-selection framework proposed by Klontzas et al. [3] to the specific context of dentomaxillofacial radiology. Rather than conducting a systematic literature review, this work provides a structured, specialty-oriented framework that maps AI study designs in panoramic imaging, CBCT, and radiomics-based investigations to the most appropriate reporting resources. Figure 1 provides a schematic overview of the typical AI research workflow in dentomaxillofacial radiology, offering the methodological context for the reporting resources discussed throughout this practical guide. This specialty-oriented perspective is particularly relevant given the diversity of imaging modalities, AI applications, and methodological considerations encountered in contemporary dentomaxillofacial imaging research [4,5,6,7,8].

Dentomaxillofacial radiology represents a particularly heterogeneous environment for AI research [4]. The main imaging modalities include panoramic radiography, cone-beam computed tomography (CBCT), computed tomography (CT), and magnetic resonance imaging (MRI), each characterized by distinct acquisition parameters, spatial resolution properties, contrast mechanisms, and diagnostic applications [5,6,7]. AI applications in this field span a wide range of tasks, including automated detection of dental and maxillofacial pathologies, segmentation of anatomical structures, prediction of treatment outcomes, and radiomics-based characterization of lesions and bone microarchitecture [4,9,10,11].

These methodological variations create important challenges for study reproducibility and interpretation. Differences in imaging acquisition, preprocessing, annotation protocols, validation strategies, and model evaluation may influence AI performance and limit comparison across studies [11]. Therefore, selecting the most appropriate reporting framework becomes increasingly challenging, particularly for hybrid AI studies that combine radiomics, machine learning, predictive modeling, and clinical validation [3].

The coexistence of multiple frameworks, including CLAIM (Checklist for Artificial Intelligence in Medical Imaging), STARD-AI (Standards for Reporting Diagnostic Accuracy Studies Using Artificial Intelligence), CONSORT-AI (Consolidated Standards of Reporting Trials–Artificial Intelligence), SPIRIT-AI (Standard Protocol Items: Recommendations for Interventional Trials–Artificial Intelligence), MI-CLAIM (Minimum Information about Clinical Artificial Intelligence Modeling), MINIMAR (MINimum Information for Medical AI Reporting), and FUTURE-AI (Fairness, Universality, Traceability, Usability, Robustness, and Explainability in Artificial Intelligence) [1,2,12,13,14,15,16]. Fairness also represents a broader methodological consideration in medical imaging AI, particularly when model performance may vary across populations or datasets [17]. Other relevant resources include TRIPOD+AI (Transparent Reporting of a Multivariable Prediction Model for Individual Prognosis or Diagnosis using Artificial Intelligence) and RQS (Radiomics Quality Score) [18,19]. These resources address different aspects of AI research, and their distinct scopes and complementary roles should be considered when selecting the most appropriate guidance for a given study.

As this article was developed as a specialty-oriented practical guide rather than a systematic review, the literature was selected through a targeted narrative search. Relevant publications were identified through PubMed, Scopus, and Google Scholar, complemented by searches of official guideline sources and reference lists of key publications. Priority was given to original reporting guidelines and methodological resources, together with representative and recent studies illustrating their application to AI research in dentomaxillofacial imaging. The literature was last updated in September 2026. No formal systematic-review inclusion or exclusion criteria were applied.

The contribution of this guide lies in translating general AI reporting recommendations into the specific methodological context of dentomaxillofacial radiology. Unlike broader guidance developed for medical imaging as a whole, it considers challenges that are particularly relevant to this field, including the heterogeneity of panoramic imaging and CBCT protocols, annotation-dependent workflows, radiomics and texture analysis, and studies that combine diagnostic, predictive, and AI modeling components. The guide also provides a practical, study-oriented approach to selecting and combining reporting resources for authors, readers, and reviewers in this specialty.

## 2. Why Reporting Guidelines Matter in Dentomaxillofacial AI Research

AI research in dentomaxillofacial radiology presents methodological challenges that differ in important ways from those encountered in other areas of medical imaging. These differences arise from the diversity of imaging modalities, the specific diagnostic tasks involved, and the structure of available datasets in this field.

A prominent characteristic of dentomaxillofacial AI studies is the reliance on retrospective datasets originally acquired for clinical purposes, often involving a limited number of scanners and heterogeneous acquisition protocols. Variations in voxel size, field of view, reconstruction algorithms, and exposure parameters in CBCT and CT, as well as sequence parameters in MRI, may influence image texture, contrast, and noise characteristics. This variability is particularly relevant in applications such as trabecular bone analysis, detection of subtle osseous changes, evaluation of temporomandibular joint structures, and radiomics-based lesion characterization. In MRI-based AI studies, especially those focused on temporomandibular joint disorders, differences in imaging sequences and multimodal integration with CBCT may further influence image interpretation, feature extraction, and AI model performance [7,11]. Without standardized reporting of these parameters, comparisons between studies and external validation become inherently difficult [1,18].

Another important source of variability lies in the definition of ground truth. Annotation is typically performed by expert radiologists and often involves subjective interpretation, especially in tasks such as detection of periapical lesions, assessment of bone remodeling, identification of anatomical variations, or delineation of regions of interest for radiomics analysis. In many studies, differences in annotator expertise, segmentation strategy, or consensus methodology are insufficiently described, limiting reproducibility and potentially introducing bias [1,18,19]. Transparent reporting of annotation protocols, rater expertise, and inter- or intraobserver agreement is therefore essential to properly interpret AI performance [1,19].

The impact of annotation uncertainty may vary according to the type of AI task being performed. In detection and diagnostic classification studies, variability primarily affects the definition of the reference standard. In segmentation tasks, differences in contour delineation may directly influence model training and performance. This issue becomes particularly important in radiomics investigations, where small variations in region-of-interest definition may considerably affect extracted features and downstream analyses. In predictive modeling studies, annotation uncertainty may additionally influence outcome definition and model calibration. Accordingly, the level of annotation detail required for transparent reporting may differ according to the intended analytical application [1,18,19].

Such challenges are particularly relevant in radiomics and other high-dimensional analytical approaches in which decisions regarding methodology during the processing of images and feature extraction can have a major impact on the study’s results. The increasing use of radiomics and high-dimensional feature analysis further amplifies methodological complexity. CBCT- and CT-based studies may involve small sample sizes, high feature-to-sample ratios, and extensive preprocessing pipelines, which can increase the risk of overfitting, data leakage, and overly optimistic performance estimates. In this context, reporting guidelines play a critical role in ensuring that feature selection, model training, validation strategies, and performance evaluation are clearly described and methodologically sound [1,9,10,11].

Moreover, the translation of AI tools into clinical dentomaxillofacial practice introduces additional considerations related to interpretability, fairness, and usability. Systems intended to support diagnosis, treatment planning, or risk assessment must demonstrate robustness across different patient populations, imaging protocols, and scanner types. Reporting frameworks that emphasize transparency, bias assessment, and intended clinical use are therefore essential to support responsible implementation [20].

Although these concepts are frequently discussed under the broader umbrella of AI generalizability, they represent different methodological challenges. External validation assesses whether a model performs well on independent datasets, whereas scanner robustness reflects its ability to maintain performance across different imaging systems and acquisition protocols. Similarly, transportability refers to the capacity of a model to generalize to new clinical environments, while patient spectrum variability relates to differences in the characteristics of the populations being studied. Clinical workflow evaluation introduces an additional layer of complexity by examining how AI systems perform under real-world conditions. Recognizing these distinctions is important because each dimension requires specific validation strategies and reporting requirements, further highlighting the value of structured reporting frameworks for documenting and communicating methodological rigor [13,16,20].

In this setting, reporting guidelines should not be viewed as administrative requirements, but as methodological instruments that address the specific vulnerabilities of AI research in dentomaxillofacial radiology. Their systematic application may improve reproducibility, facilitate peer review, and support the responsible integration of artificial intelligence into clinical practice.

With this rationale established, the following sections provide an overview of the major AI reporting guidelines currently available and discuss their applicability to representative dentomaxillofacial imaging research scenarios.

## 3. Overview of AI Reporting Resources Relevant to Dentomaxillofacial Radiology

Although the resources discussed in this section are presented together because they support the conduct, reporting, evaluation, and implementation of AI research, they were developed for different methodological purposes and should not be regarded as interchangeable. Some focus on reporting completeness for specific study designs, whereas others emphasize methodological quality, reproducibility, or broader principles for trustworthy AI development and clinical implementation. The following sections summarize the main characteristics of these complementary resources and discuss their relevance to dentomaxillofacial radiology.

### 3.1. CLAIM

CLAIM was developed to improve transparency and reproducibility in AI-based medical imaging research by providing a structured reporting checklist for reporting model development and validation studies [1]. Unlike broader methodological recommendations, CLAIM focuses specifically on AI workflows and encourages detailed reporting of dataset composition, preprocessing strategies, annotation procedures, data partitioning, model architecture, training methodology, and performance evaluation.

In dentomaxillofacial radiology, CLAIM is particularly relevant because many studies involve heterogeneous retrospective datasets, multiple acquisition protocols, and operator-dependent annotation processes. Applications such as automated detection of periapical lesions, segmentation of mandibular canal, classification of maxillofacial pathologies, and CBCT-based radiomics analyses all require careful description of imaging parameters and annotation procedures. Without such transparency, comparisons between studies become difficult and reproducibility may be compromised.

Another important aspect of CLAIM is its emphasis on data handling and potential sources of bias. This is especially valuable in dentomaxillofacial imaging, where AI performance may be strongly influenced by differences in scanner manufacturer, voxel size, field of view, image reconstruction, and annotation strategy. Consequently, CLAIM serves as the primary reporting guideline for most non-interventional AI investigations in panoramic radiography, CBCT, CT, and MRI-based dentomaxillofacial studies. The relationship between study designs and the reporting resources discussed in this guide is summarized in Table 1.

### 3.2. CONSORT-AI and SPIRIT-AI

CONSORT-AI and SPIRIT-AI extend traditional clinical trial reporting standards to investigations involving AI-based interventions [3,12,13]. While most AI studies in dentomaxillofacial radiology remain retrospective or technically oriented, prospective and interventional evaluations under real clinical conditions are still limited. This gap is particularly relevant for AI systems intended for diagnostic support, treatment planning assistance, and workflow optimization, for which prospective evaluation will be important before routine clinical implementation.

These reporting guidelines address aspects that are often underreported in AI studies, including participant recruitment, intended users, integration of AI systems into clinical workflows, randomization procedures, and evaluation of potential harms associated with AI-assisted decision-making. SPIRIT-AI is primarily intended for reporting clinical trial protocols, whereas CONSORT-AI focuses on reporting completed AI intervention trials.

In practical terms, these reporting guidelines become particularly important when AI systems are evaluated under real clinical conditions rather than purely technical environments. For example, a study investigating whether AI-assisted interpretation of panoramic radiographs improves diagnostic consistency among clinicians would require assessment of factors that extend beyond algorithm performance alone. Issues such as usability, interaction between clinicians and AI systems, and potential workflow impact become equally relevant.

### 3.3. STARD-AI and TRIPOD+AI

STARD-AI and TRIPOD+AI extend the original STARD and TRIPOD reporting guidelines by incorporating methodological recommendations that address challenges specific to AI-based studies. While STARD-AI focuses on diagnostic accuracy investigations involving AI-centered index tests, TRIPOD+AI provides updated guidance for studies developing or evaluating clinical prediction models using regression or machine learning methods. Both reporting guidelines retain the core methodological principles of their predecessors while introducing additional recommendations related to AI workflows, dataset management, model development, validation, transparency, fairness, and reproducibility [2,18].

In dentomaxillofacial radiology, STARD-AI is particularly relevant for studies evaluating the diagnostic accuracy of AI systems, such as the detection of periapical lesions, root fractures, cystic lesions, temporomandibular joint abnormalities, or other pathologies using panoramic radiography or CBCT. Beyond the traditional STARD recommendations, STARD-AI emphasizes AI-specific aspects including dataset provenance, annotation procedures, preprocessing, algorithm development, external evaluation, fairness, and transparency [2].

TRIPOD+AI is particularly applicable when AI methods are used to develop or evaluate prediction models. Examples in dentomaxillofacial radiology include radiomics-based prediction of lesion behavior, estimation of treatment outcomes, or models integrating imaging-derived features with clinical variables. Compared with the original TRIPOD statement, TRIPOD+AI introduces additional recommendations addressing machine learning methods, fairness, open science practices, data and code sharing, model reproducibility, and transparent reporting of model development and evaluation. The TRIPOD+AI statement supersedes the original TRIPOD 2015 reporting guideline for AI-based prediction models [18].

Many contemporary dentomaxillofacial AI studies combine diagnostic accuracy assessment, prediction modeling, and radiomics in the same workflow. In these situations, a single reporting guideline may not always be sufficient. Depending on the principal objective of the study, complementary use of STARD-AI, TRIPOD+AI, CLAIM, and RQS may provide a more comprehensive approach to study reporting and methodological evaluation [1,2,18,19].

### 3.4. MI-CLAIM and MINIMAR

MI-CLAIM and MINIMAR were developed to strengthen transparency and reproducibility in clinical AI modeling [15,16]. Compared with CLAIM, which primarily focuses on reporting AI studies in medical imaging, these reporting resources place greater emphasis on data provenance, reproducibility across institutions, and transparency of analytical pipelines.

These aspects are especially important in dentomaxillofacial radiology because many studies rely on relatively small institutional datasets and heterogeneous imaging protocols. Multicenter studies involving panoramic radiography or CBCT often face significant variability in acquisition parameters, annotation standards, and patient populations, all of which may influence AI performance.

MI-CLAIM and MINIMAR also encourage researchers to explicitly address issues such as distribution shift, missing data, model reproducibility, and real-world applicability. These aspects are recurrently underreported in AI imaging studies despite their importance for clinical implementation.

### 3.5. FUTURE-AI

FUTURE-AI differs from traditional reporting guidelines because it focuses on broader principles for the trustworthy development, evaluation, and deployment of AI systems [14]. Rather than functioning as a reporting guideline, the framework emphasizes concepts such as fairness, robustness, explainability, traceability, and usability throughout the entire AI lifecycle.

These principles are particularly relevant in dentomaxillofacial radiology because imaging variability can affect AI performance. Differences in scanner manufacturer, acquisition settings, reconstruction algorithms, and patient demographics may all influence model generalizability. As a result, studies that report excellent performance under highly controlled conditions may fail when applied to external datasets or routine clinical environments.

FUTURE-AI therefore encourages researchers to consider issues that extend beyond technical accuracy, including bias assessment, robustness across scanners and populations, explainability of predictions, and transparency regarding intended clinical use. These concepts become increasingly important as AI systems move closer to real-world implementation.

In dentomaxillofacial radiology, however, evidence from prospective clinical deployment remains limited. FUTURE-AI should therefore be viewed primarily as guidance for anticipating requirements related to trustworthiness and implementation as AI systems progress toward prospective and real-world evaluation.

### 3.6. RQS

The RQS was specifically developed to assess methodological rigor in radiomics investigations [19]. Unlike broader AI reporting guidelines, RQS is a methodological quality assessment tool that focuses on aspects directly related to feature extraction, segmentation reproducibility, validation strategies, and clinical utility.

Radiomics has become increasingly prominent in dentomaxillofacial radiology, particularly in CBCT and CT-based analyses of bone microarchitecture, lesion heterogeneity, and temporomandibular joint alterations. MRI-based radiomics has also been explored in soft tissue characterization and inflammatory joint assessment, particularly in the evaluation of temporomandibular joint disorders, where MRI provides complementary information to CBCT by depicting soft tissue structures that cannot be adequately assessed with osseous imaging alone [7]. The growing integration of MRI and CBCT in multimodal AI and radiomics studies has further expanded the potential for imaging biomarkers that combine structural and soft tissue information [11]. However, radiomic features are highly sensitive to acquisition and reconstruction parameters, making standardization especially important.

RQS encourages practices such as standardized imaging protocols, test–retest analysis, feature robustness evaluation, and independent validation. At the same time, it has important limitations when used alone in AI-driven studies. RQS does not comprehensively address model architecture, data partitioning, training methodology, or algorithm transparency. For this reason, radiomics-based AI investigations in dentomaxillofacial imaging should generally combine RQS with AI reporting guidelines such as CLAIM and, when predictive modeling is involved, with TRIPOD+AI. Table 2 presents some of the methodological aspects that are still inconsistently reported in dentomaxillofacial AI studies, highlighting how these limitations may affect reproducibility, interpretation of results, and potential clinical applicability, as well as the reporting frameworks that can help address them.

## 4. How to Select and Combine Reporting Guidelines in Dentomaxillofacial AI Studies

The proposed adaptation was founded on methodological features that are especially prevalent in dentomaxillofacial AI research. These features include the dominance of CBCT and panoramic imaging, the widespread use of radiomics and texture analysis, the dependence on retrospective datasets, the workflow that is reliant on annotation, and the increasing use of predictive and diagnostic accuracy models. These characteristics were used to contextualize and tailor the application of existing reporting frameworks to dentomaxillofacial imaging research. The mappings presented in this article were developed by the authors based on the intended scope of existing reporting guidelines and their application to representative AI study designs in dentomaxillofacial radiology. For each study design, the main reporting resource was identified according to its direct alignment with the major objective and methodological focus of the investigation. Additional resources were considered when they addressed relevant components not fully covered by the main resource, such as radiomics quality, prediction modeling, reproducibility, trustworthy AI principles, or clinical implementation. The resulting pairings therefore reflect the complementary functions of these resources rather than a hierarchy of importance. They are intended to provide practical methodological guidance for readers and reviewers. Because they have not been formally validated through consensus methods or empirical evaluation, they should be interpreted as a conceptual guide rather than prescriptive recommendations.

When multiple reporting resources are applicable, the most relevant resource should be selected according to the central objective of the study, while additional resources may be used to address other relevant methodological components. In this approach, reporting frameworks are viewed as additive tools rather than competing ones, with each guideline contributing to different aspects of study design, validation, reporting transparency, or clinical translation. Rather than replacing existing reporting standards or serving as a validated assessment tool, the proposed framework is intended to provide a specialty-oriented perspective that may help readers, reviewers, and researchers navigate the methodological complexity of contemporary dentomaxillofacial AI studies.

Choosing an appropriate reporting guideline in dentomaxillofacial radiology is rarely an easy choice. Although each framework was originally developed for a specific type of investigation, contemporary AI studies often combine multiple analytical components into a single workflow [3,19]. In practice, many investigations simultaneously incorporate radiomics, machine learning classification, predictive modeling, and clinical validation. This overlap creates uncertainty not only for authors preparing manuscripts, but also for reviewers attempting to evaluate methodological quality and reproducibility [3,17,18].

**Figure 2:**
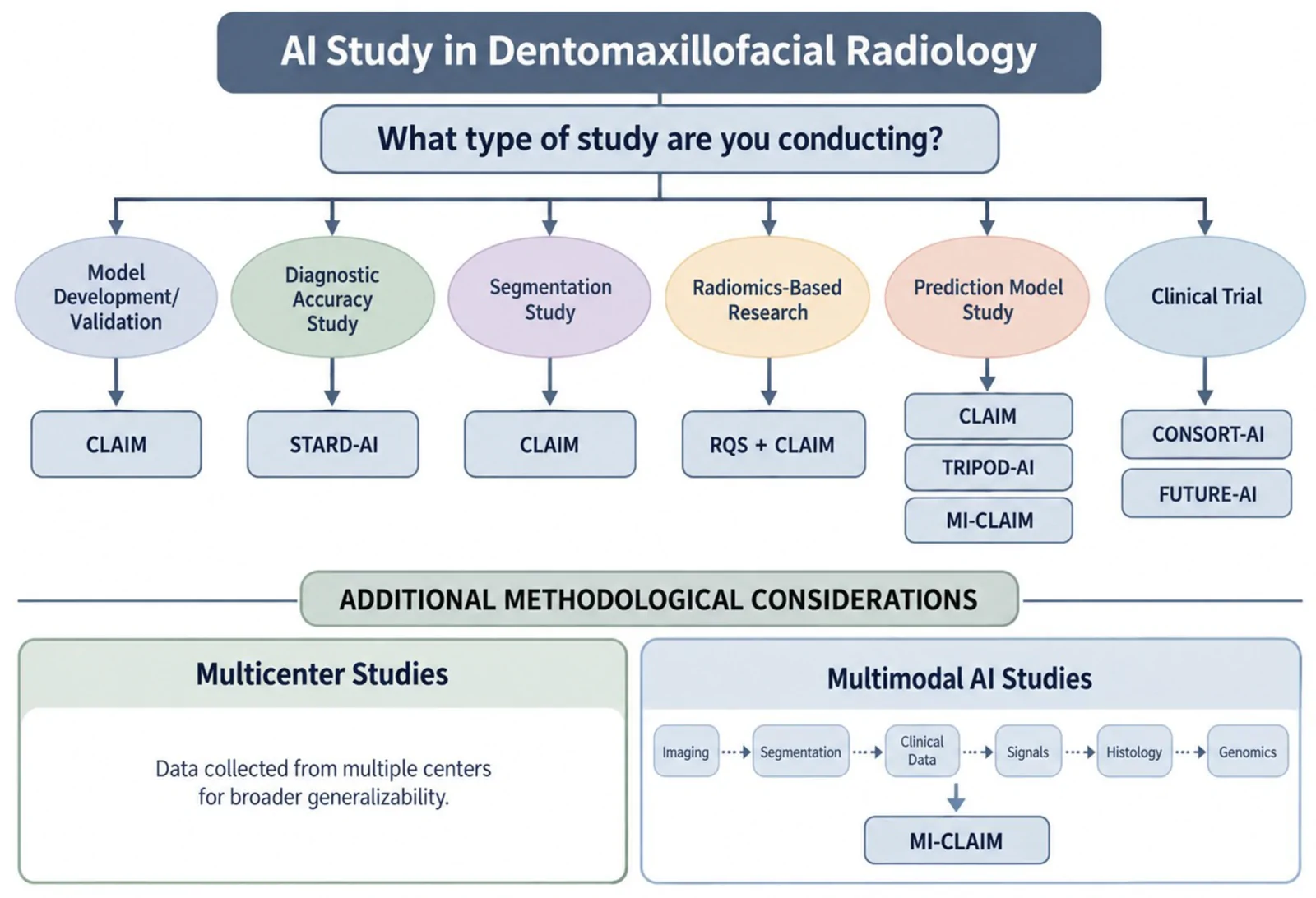
Practical guide for selecting AI reporting resources in dentomaxillofacial radiology. The figure illustrates a conceptual approach for selecting reporting resources according to the study type and methodological focus, while highlighting additional considerations for multicenter and multimodal AI studies. The proposed pathways are intended as practical guidance and should not be interpreted as rigid rules or a validated decision algorithm.

Figure 2 summarizes this approach, showing how reporting resources can be selected according to the main objective of an AI study and complemented when additional methodological or clinical considerations are involved [3].

For studies centered on AI model development or technical validation, CLAIM provides the most comprehensive foundation because it emphasizes transparent reporting of dataset composition, preprocessing strategies, annotation procedures, data partitioning, model architecture, training methodology, and performance evaluation [1]. These elements are particularly important in dentomaxillofacial radiology, where differences in scanner manufacturer, voxel size, field of view, reconstruction algorithms, and annotation strategy may influence AI performance.

However, many AI investigations in dentomaxillofacial imaging extend beyond simple classification tasks. A CBCT-based study evaluating radiomic features to predict progression of periapical lesions, for example, simultaneously involves feature extraction, predictive modeling, and machine learning classification. In such hybrid designs, relying on a single reporting framework is usually insufficient.

**Figure 3:**
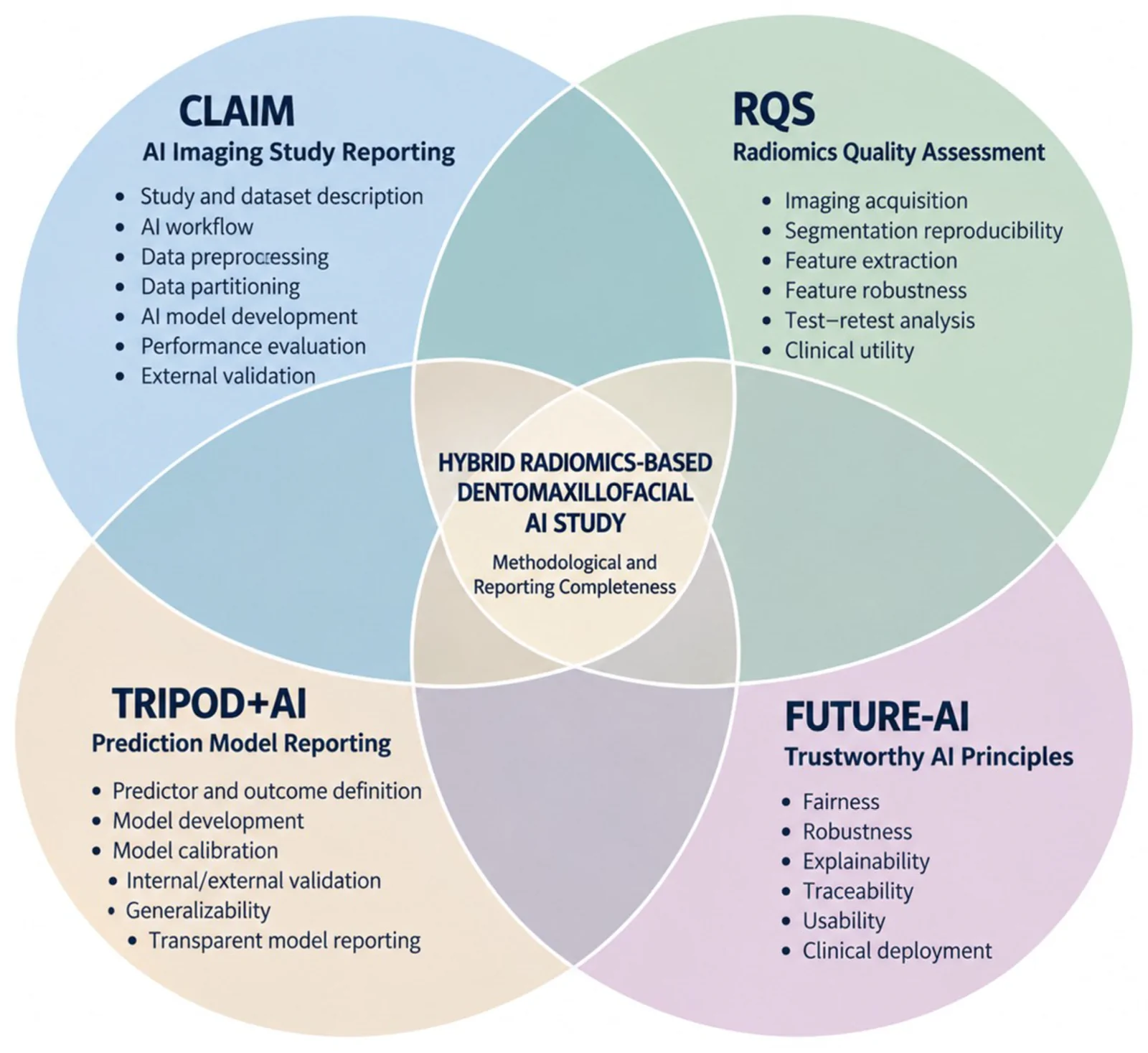
Complementary roles of AI reporting resources in hybrid radiomics-based dentomaxillofacial studies. The figure illustrates how CLAIM, RQS, TRIPOD+AI, and FUTURE-AI address distinct but complementary methodological and reporting aspects of hybrid AI investigations. Their overlap represents how these resources may contribute to methodological and reporting completeness when different analytical components are combined within the same study.

In these situations, the complementary use of multiple reporting resources may provide a more comprehensive approach to study reporting. RQS contributes radiomics-specific rigor by addressing segmentation reproducibility, feature robustness, and validation strategy [19]. CLAIM complements this by ensuring transparent reporting of AI workflow development and model evaluation [1]. When prediction of clinical outcomes or lesion behavior is included, TRIPOD+AI principles become equally important for reporting model calibration, validation procedures, and generalizability [18]. Figure 3 conceptually illustrates how CLAIM, RQS, TRIPOD+AI, and FUTURE-AI provide complementary guidance for different methodological and reporting aspects of hybrid radiomics-based AI studies.

A similar overlap may occur in diagnostic accuracy studies. AI systems developed for detecting root fractures, periapical lesions, cysts, or anatomical variations in panoramic radiography and CBCT are often presented primarily as classification models, yet they also function as diagnostic accuracy investigations. In these cases, STARD-AI principles help ensure appropriate reporting of patient selection, reference standards, and clinically meaningful performance metrics [2], while CLAIM provides additional guidance regarding annotation procedures, preprocessing steps, and AI-specific evaluation methods [1].

The need for combined frameworks becomes even more evident in multicenter and clinically oriented investigations. Studies integrating imaging-derived features with demographic, clinical, or treatment-related variables often require simultaneous consideration of CLAIM, TRIPOD+AI, and frameworks such as MI-CLAIM or MINIMAR [14,15]. These recommendations are particularly relevant in dentomaxillofacial radiology because many datasets are retrospective, institution-specific, and highly heterogeneous in terms of acquisition protocols.

Prospective or interventional investigations introduce additional reporting demands. AI systems evaluated as decision-support tools for implant planning, orthodontic assessment, temporomandibular joint analysis, or lesion detection may require CONSORT-AI or SPIRIT-AI to ensure transparent reporting of participant flow, intended users, workflow integration, and potential harms associated with AI-assisted decision-making [12,13]. Beyond this, broader issues related to fairness, explainability, robustness, and usability should be considered through frameworks such as FUTURE-AI [14,21].

From a reviewer perspective, overlapping frameworks should not be interpreted as a methodological weakness, but rather as a reflection of the increasing complexity of AI investigations. Reviewers should therefore evaluate whether the selected reporting standards adequately represent the analytical components present in the study. A radiomics investigation lacking feature reproducibility analysis, a predictive model without external validation, or an AI classification study with poorly described annotation protocols should all be interpreted cautiously, regardless of high reported performance metrics [18,19,20]. These concerns are not merely theoretical. Recent reviews of dental AI research have documented substantial variability in reporting quality, external validation practices, and methodological transparency, particularly in studies involving diagnostic imaging and deep learning models [4,8,9].

Several practical “red flags” may help reviewers and readers identify potentially weak AI studies in dentomaxillofacial radiology. Although high performance metrics may initially appear convincing, methodological limitations often become evident when important aspects of study design, reproducibility, and validation are examined more carefully [8,20].

Another concern relates to the absence of external validation using independent datasets. Many AI studies rely exclusively on retrospective institutional data, which may limit generalizability, particularly in dentomaxillofacial imaging where differences in CBCT systems, acquisition protocols, and reconstruction parameters can extensively influence image characteristics and model performance [1,21]. Similarly, insufficient reporting of imaging acquisition details, preprocessing methods, or feature selection strategies may compromise reproducibility and make comparisons between studies difficult [1,19].

Another important warning sign is the inadequate description of ground truth generation and annotator expertise. In dentomaxillofacial radiology, many AI tasks depend on subjective interpretation by experienced radiologists, especially in the detection of periapical lesions, assessment of bone remodeling, segmentation of anatomical structures, or delineation of radiomic regions of interest. When studies fail to clearly report annotation protocols, consensus methodology, or inter- and intraobserver agreement, interpretation of AI performance becomes significantly less reliable [1,20].

Reproducibility can become even more challenging when studies provide limited information about data availability, source code, or implementation details. Although sharing imaging datasets is not always possible because of privacy, ethical, or regulatory constraints, transparent reporting of data access, code availability, model parameters, and implementation procedures can greatly improve the ability of other researchers to understand, reproduce, and independently verify the reported findings. When this information is missing or only partially described, readers and reviewers may find it difficult to fully assess the reliability and reproducibility of the study [1,15,16].

Readers/Reviewers should also be cautious when highly complex models are developed using relatively small datasets or when studies rely heavily on isolated performance metrics without adequate clinical interpretation. Excellent accuracy or AUC (Area Under the Curve) values do not necessarily imply clinical usefulness, particularly when models have not been tested under independent or real-world conditions [8,18,20]. In diagnostic applications, sensitivity and specificity are often more informative than AUC alone because they directly reflect the ability of a model to correctly identify positive and negative cases. Their interpretation becomes even more meaningful when accompanied by confidence intervals, which provide insight into the precision and stability of performance estimates. Depending on the intended clinical application, different balances between sensitivity and specificity may be required, making these metrics particularly relevant when assessing the potential clinical utility of AI systems [2,20].

Furthermore, in radiomics-based investigations, lack of reproducibility analysis or insufficient attention to feature robustness may further increase the risk of overfitting and unstable results [11,19].

Taken together, these potential indicators of weakness do not automatically invalidate an AI investigation, but they should encourage more careful interpretation of reported findings. Studies that transparently describe imaging protocols, annotation procedures, preprocessing pipelines, validation strategies, and methodological limitations are considerably more likely to provide reproducible and clinically meaningful results [1,8,14].

## 5. Practical Guidance for Readers and Reviewers

The conceptual approach presented in the previous section is intended to support the practical recommendations that follow. The following points are designed to help readers and reviewers critically assess the reporting and methodological quality of AI studies in dentomaxillofacial radiology, using the principles summarized in Figure 2 as a practical reference.

### 5.1. Identify the Primary Objective of the Study

The first step is to determine whether the investigation primarily focuses on AI model development, diagnostic accuracy, radiomics analysis, predictive modeling, or clinical validation. This distinction is important because the most appropriate reporting framework depends largely on the dominant methodological component of the study [3].

### 5.2. Evaluate Whether the Selected Guideline Matches the Study Design

Studies centered on AI workflow development and technical validation should generally follow CLAIM [1], whereas diagnostic accuracy investigations are better aligned with STARD-AI principles [2]. Predictive models involving clinical outcomes or prognostic estimation should incorporate TRIPOD+AI recommendations [18], while radiomics-based investigations should additionally consider RQS [19].

### 5.3. Assess Whether Complementary Frameworks Are Necessary

Many dentomaxillofacial AI studies combine multiple analytical components in the same workflow. Recent examples include studies on the evaluation of periapical status and dental caries using AI-assisted radiographic assessment [22], automated mandibular canal and anterior loop segmentation on CBCT images [23], detection of temporomandibular joint osteoarthritis on panoramic radiographs [24], and broader AI-based oral disease diagnosis or imaging assessment models [25]. Other studies further illustrate the diversity of AI applications in dentomaxillofacial imaging, including the development of annotated imaging datasets and deep learning approaches for tooth and anatomical structure segmentation using panoramic radiography and CBCT [26,27,28]. These studies illustrate how contemporary AI investigations often extend beyond a single methodological category. For example, studies aimed at detecting periapical lesions in CBCT or panoramic radiographs may function both as AI classification models and diagnostic accuracy investigations, making the combined use of CLAIM and STARD-AI particularly relevant [1,2]. Similarly, automated segmentation studies involving structures such as the mandibular canal rely heavily on annotation quality, data partitioning, and validation procedures, making CLAIM an important reporting framework. In radiomics or TMJ-related investigations, RQS may help address feature robustness and segmentation reproducibility, while CLAIM and TRIPOD+AI provide complementary guidance when predictive modeling is involved [11,18,19]. These examples illustrate how reliance on a single framework may be insufficient in contemporary dentomaxillofacial AI studies.

### 5.4. Critically Analyze Transparency and Reproducibility

Readers and reviewers should assess whether imaging acquisition parameters, preprocessing methods, annotation protocols, feature selection strategies, and validation procedures are described with sufficient detail to allow reproducibility [1,20,21]. Lack of external validation or inadequate reporting of ground truth generation should be interpreted cautiously.

### 5.5. Interpret Performance Metrics Within a Clinical Context

High accuracy or AUC values alone do not necessarily indicate clinical usefulness. Reported results should be interpreted in relation to dataset heterogeneity, external testing, intended clinical application, and comparison with expert performance [8,20].

### 5.6. Consider Potential Limitations and Sources of Bias

Differences in scanner type, acquisition protocols, retrospective datasets, and annotator variability may significantly affect AI performance in dentomaxillofacial imaging. Frameworks such as FUTURE-AI may help readers and reviewers identify concerns related to fairness, robustness, and generalizability [14,17].

The recommendations presented in this section are not intended to function as rigid scoring criteria or decision rules. Instead, they are designed to help readers and reviewers recognize situations in which reporting may be incomplete or where additional methodological detail may be needed. The importance of any reporting limitation should be considered in light of the study design, intended application, and reporting framework adopted by the authors.

Rather than replacing critical judgment, these recommendations are intended to provide a practical framework for evaluating the strengths and limitations of AI studies in a more consistent and informed manner.

Together, these practical recommendations may facilitate more consistent critical appraisal of AI studies and encourage more transparent, reproducible, and clinically meaningful research in dentomaxillofacial radiology.

Table 3 summarizes these practical considerations as a concise reference for readers and reviewers when evaluating dentomaxillofacial AI studies.

## 6. Limitations

This article has some limitations. It was developed as a specialty-oriented practical guide rather than a systematic review, and the literature was selected through a targeted narrative approach without a formal systematic search or predefined inclusion and exclusion criteria. The sources included should therefore be considered representative rather than exhaustive. The recommendations presented in this guide reflect a conceptual adaptation of existing reporting resources to the context of dentomaxillofacial radiology. The proposed mappings are based on the authors’ conceptual evaluation and have not been independently validated through expert review, consensus methods, or empirical application to a representative sample of published studies. This should be considered when interpreting the proposed pairings, which are intended to provide practical guidance rather than validated or prescriptive recommendations. Future studies may help evaluate and refine this approach through expert consensus and application to representative samples of published AI studies.

## 7. Conclusions

AI research in dentomaxillofacial radiology involves different study designs, each with its own reporting and methodological needs. This practical guide clarifies how existing reporting resources can be applied according to the purpose and characteristics of each investigation, while recognizing that some studies may benefit from more than one resource. The proposed approach is intended to support, rather than prescribe, methodological decisions and to provide researchers, readers, and reviewers with a practical reference when evaluating AI studies in this field. As AI moves closer to clinical application, clear and appropriate reporting will remain important for understanding how reliably these tools perform and how well they may translate into clinical practice.

## Figures and Tables

**Table 1:** Alignment of Common AI Study Designs in Dentomaxillofacial Radiology with Reporting Guidelines.

Study Type	Typical Imaging Modality	Main Reporting Resource	Additional Relevant Resource(s)	Primary Reporting Focus
AI model development	CT, MRI, CBCT, panoramic radiography	CLAIM [1]	FUTURE-AI [14]	Transparency, reproducibility, and reporting of AI workflows
Diagnostic accuracy	CT, MRI, CBCT, panoramic radiography	STARD-AI [2]	CLAIM [1]	Reference standards, patient selection, and clinically meaningful performance metrics
Radiomics analysis	CT, MRI, CBCT	RQS [19]	CLAIM [1]	Feature robustness, segmentation reproducibility, and validation strategy
Predictive modeling	CT, MRI, CBCT	TRIPOD+AI [18]	MI-CLAIM [15]	Model calibration, validation, and generalizability
Clinical trial	Multiple dentomaxillofacial imaging modalities	CONSORT-AI [12]	FUTURE-AI [14]	Clinical integration, safety, and workflow impact
Trial protocol	Multiple dentomaxillofacial imaging modalities	SPIRIT-AI [13]	—	Transparent protocol design and intended clinical use

Note: The proposed pairings are intended as practical guidance rather than rigid or prescriptive rules. The main reporting resource reflects the main objective and methodological focus of each study type, while additional resources may be considered according to the specific characteristics of the investigation. Abbreviations: AI (artificial intelligence); CBCT (cone-beam computed tomography); CT (computed tomography); MRI (magnetic resonance imaging); CLAIM (Checklist for Artificial Intelligence in Medical Imaging); STARD-AI (Standards for Reporting Diagnostic Accuracy Studies Using Artificial Intelligence); RQS (Radiomics Quality Score); TRIPOD+AI (Transparent Reporting of a Multivariable Prediction Model for Individual Prognosis or Diagnosis Using Artificial Intelligence); MI-CLAIM (Minimum Information about Clinical Artificial Intelligence Modeling); CONSORT-AI (Consolidated Standards of Reporting Trials–Artificial Intelligence); SPIRIT-AI (Standard Protocol Items: Recommendations for Interventional Trials–Artificial Intelligence); FUTURE-AI (Fairness, Universality, Traceability, Usability, Robustness, and Explainability in Artificial Intelligence).

**Table 2:** Key Reporting Elements Requiring Attention in Dentomaxillofacial AI Studies.

Reporting Element	Potential Issue	Relevant Guideline	Potential Reader/Reviewer Concern
Scanner parameters	Incomplete description of imaging acquisition and reconstruction parameters	CLAIM [1], RQS [19]	Limited reproducibility and reduced generalizability [8,19]
Annotation protocol	Unclear annotation methodology or insufficient rater description	CLAIM [1]	Potential annotation bias and inconsistent reference standards [1,8,19]
Data splitting strategy	Inadequate separation of training and testing datasets	CLAIM [1]	Risk of data leakage and overestimated performance [1,8,19]
External validation	Lack of independent or multicenter testing	CLAIM [1], RQS [19]	Limited clinical applicability and poor generalizability [8,18]
Bias and fairness	Insufficient evaluation across populations or scanners	FUTURE-AI [14]	Reduced robustness and hidden algorithmic bias [14,17,21]
Clinical integration	Limited discussion of intended clinical use and workflow impact	CONSORT-AI [12], FUTURE-AI [14]	Unclear clinical relevance and translational value [8,14]
Data and code availability	Lack of access to datasets, source code, or implementation details	CLAIM [1], MI-CLAIM [15], MINIMAR [16]	Limited reproducibility and inability to independently verify findings [1,15,16]

Note: The reporting elements presented in Table 2 reflect recurring concerns identified in methodological discussions and reviews of AI research [4,8,9]. They are included as representative reporting considerations and should not be interpreted as quantitative estimates of their prevalence in the literature.

**Table 3:** Practical Checklist for Readers and Reviewers of Dentomaxillofacial AI Studies.

Assessment Point	Key Question for Readers/Reviewers	Relevant Reporting Resource(s)
Study objective	Is the primary objective of the study clearly defined?	CLAIM; STARD-AI; TRIPOD+AI; RQS
Study design and reporting	Does the selected reporting resource match the study design and analytical objective?	CLAIM; STARD-AI; TRIPOD+AI; CONSORT-AI; SPIRIT-AI
Complementary methodological components	Does the study include additional components that may require complementary reporting resources?	CLAIM; RQS; TRIPOD+AI; MI-CLAIM; MINIMAR
Transparency and reproducibility	Are imaging acquisition, preprocessing, annotation, data partitioning, and validation procedures adequately described?	CLAIM; RQS; MI-CLAIM; MINIMAR
Performance and clinical relevance	Are performance metrics interpreted in relation to the intended clinical application and external validation?	STARD-AI; TRIPOD+AI; CONSORT-AI
Bias and generalizability	Are potential sources of bias, scanner variability, fairness, robustness, and generalizability considered?	FUTURE-AI; CLAIM

## Data Availability

No new data were created or analyzed in this study. Data sharing is not applicable to this article.
